# Human-Induced Pluripotent Stem Cells (iPSCs) for Disease Modeling and Insulin Target Cell Regeneration in the Treatment of Insulin Resistance: A Review

**DOI:** 10.3390/cells14151188

**Published:** 2025-08-01

**Authors:** Sama Thiab, Juberiya M. Azeez, Alekya Anala, Moksha Nanda, Somieya Khan, Alexandra E. Butler, Manjula Nandakumar

**Affiliations:** Royal College of Surgeons in Ireland Bahrain, Busaiteen 15503, Bahrain; 21202342@rcsi-mub.com (S.T.); jazeez@rcsi-mub.com (J.M.A.); 20203265@rcsi-mub.com (A.A.); 21202073@rcsi-mub.com (M.N.); 20205059@rcsi-mub.com (S.K.)

**Keywords:** human-induced pluripotent stem cells, insulin resistance, diabetes mellitus, iPSC-derived insulin target cells, iPSC differentiation

## Abstract

Diabetes mellitus, both type 1 (T1D) and type 2 (T2D), has become the epidemic of the century and a major public health concern given its rising prevalence and the increasing adoption of a sedentary lifestyle globally. This multifaceted disease is characterized by impaired pancreatic beta cell function and insulin resistance (IR) in peripheral organs, namely the liver, skeletal muscle, and adipose tissue. Additional insulin target tissues, including cardiomyocytes and neuronal cells, are also affected. The advent of stem cell research has opened new avenues for tackling this disease, particularly through the regeneration of insulin target cells and the establishment of disease models for further investigation. Human-induced pluripotent stem cells (iPSCs) have emerged as a valuable resource for generating specialized cell types, such as hepatocytes, myocytes, adipocytes, cardiomyocytes, and neuronal cells, with diverse applications ranging from drug screening to disease modeling and, importantly, treating IR in T2D. This review aims to elucidate the significant applications of iPSC-derived insulin target cells in studying the pathogenesis of insulin resistance and T2D. Furthermore, recent differentiation strategies, protocols, signaling pathways, growth factors, and advancements in this field of therapeutic research for each specific iPSC-derived cell type are discussed.

## 1. Introduction

Insulin, an anabolic peptide hormone secreted by pancreatic beta cells, is essential for glucose homeostasis and survival. It regulates glucose levels in various organs, primarily the liver, skeletal muscle, and adipose tissue [1]. In the liver, insulin promotes glucose storage as glycogen, and in skeletal muscle and adipose tissue, insulin stimulates glucose transport via the glucose transport protein, GLUT4. Brain insulin signaling participates in the regulation of brain homeostatic processes and neuropathological processes, including cognitive decline and Alzheimer’s disease. In all these target tissues, insulin functions by binding to specific receptors located on the membranes of target cells [2,3].

Disruption in insulin production or function can lead to the dysregulation of blood glucose, ultimately resulting in diabetes. Of the two major types, type 1 diabetes mellitus (T1D) is caused by a deficiency in insulin secretion due to autoimmune destruction of beta cells, whereas T2D is characterized by a combination of insulin resistance (IR) with an inadequate compensatory insulin secretory response due to loss of beta cells through apoptosis [4,5].

**Figure 1:**
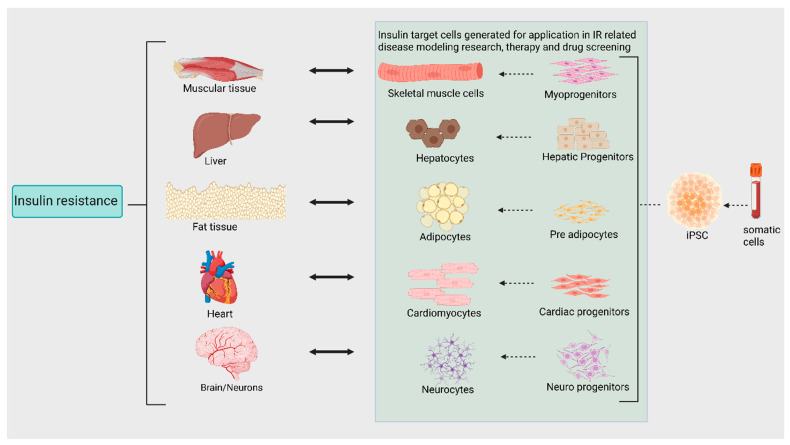
Overview of insulin resistance (IR) in key tissues (muscle, liver, fat, heart, and brain) and the generation of corresponding insulin target cells from induced pluripotent stem cells (iPSCs). Somatic cells are reprogrammed to iPSCs, differentiated into progenitors, and then cultured into mature cell types for IR research, therapy, and drug screening.

IR affects not only classical insulin target tissues (liver, skeletal muscle, and adipose tissues) but also the brain, cardiomyocytes, and nephrons. In the liver, IR leads to impaired glucose output and fatty acid metabolism, and in skeletal muscle, it hinders glycogen synthesis and protein catabolism. In adipocytes, it inhibits lipoprotein lipase activity, and in the brain, it is linked to cognitive impairment and neurodegenerative disease. IR in cardiomyocytes can lead to lipotoxicity and cardiac dysfunction, and in nephrons, it worsens glucose intolerance [6,7]. To better understand the pathogenesis and to develop effective treatments against IR, further studies are needed to investigate the specific pathogenic mechanisms in these tissues and identify drug targets and therapies (Figure 1). Induced pluripotent stem cells (iPSCs) offer a powerful tool for investigating IR and its associated conditions [8].

Here, we present a comprehensive review of the latest research utilizing insulin target cells derived from induced pluripotent stem cells (iPSCs). Specifically, we focus on iPSC-derived hepatocytes, skeletal muscle cells, adipocytes, cardiomyocytes, and neural cells for investigating and treating IR, T2D, and related complications. Further, we emphasize the utilization of iPSC-derived cells in disease modeling, drug screening, and therapeutic applications and indicate where gaps in the research exist.

## 2. Reprogramming Somatic Cells into iPSCs

Several stages are required for the differentiation of insulin target cells from iPSCs, all of which begin with the generation of iPSCs from adult somatic cells using pluripotency-inducing transcription factors [8]. While a variety of adult somatic cell types exist as precursors for iPSC generation, fibroblasts and peripheral blood mononuclear cells (PBMCs) have proven to be the most convenient sources of somatic cells, due to easy availability and viability in culture [9].

While many methods of reprogramming exist, direct reprogramming of adult somatic cells by transduction to generate iPSCs via four main transcription factors (Yamanaka factors) has revolutionized the field of stem cell research since its discovery [10]. Transduction of adult human dermal fibroblasts with these four transcription factors, *octamer-binding transcription factor (OCT3/4)*, *sex-determining region Y-box 2 (SOX2)*, *cellular Myc (c-Myc) and Kruppel-like factor 4 (KLF4)*, via a retroviral or lentiviral vector and transfection with an mRNA vector has proven to be an effective strategy for generating pluripotent stem cells from somatic cells [11,12]. Transduction efficiency, safety, protocol optimization, and availability of an integration-free product are key elements considered in vector selection [12].

After reprogramming, iPSC colonies are selected and expanded and then characterized and validated for their ability to differentiate into the three germ layers before being considered for directed differentiation.

## 3. iPSC-Derived Hepatocytes

### 3.1. Stages, Signaling Molecules, and Growth Factor

#### 3.1.1. Definitive Endoderm Induction

Hepatocytes are derived from endodermal cells during embryonic development [13]. When differentiating iPSCs into hepatocytes in vitro, specific signaling molecules called morphogens induce the patterning process, leading to definitive endoderm (DE) formation [14]. Transforming growth factor-β (TGFβ) superfamily members, including activin A, TGFβ, bone morphogenetic protein 4 (BMP4), inhibins, and nodal proteins play crucial roles in endoderm formation prior to hepatic specification [15]. Activin A is a key inducer of iPSC differentiation into DE, both in vivo and in vitro [16,17,18]. A recent study reported that culturing iPSCs on a gold nanoparticle gradient surface functionalized with activin A yielded optimal results for endoderm induction, surpassing the conventional protocol when using a high concentration of activin A (100 ng/mL) [19]. Typically, iPSCs are incubated with human activin A and fetal bovine serum (FBS) for ~5 days, leading to differentiation into the anterior primitive streak followed by DE formation [19].

In addition to activin A, various small molecules can be employed in combination to enhance DE differentiation, including wingless-related integration site (Wnt) activators (CHIR99021), BMP activators (BMP4), and fibroblast growth factors (FGF2) [19,20]. At this stage, the differentiated iPSCs express DE markers, such as sex-determining region Y-box 17 (SOX17) and GATA-binding factor 4 (GATA4) [14], which drive their subsequent differentiation into immature hepatocytes or hepatoblasts.

Given the costs of cytokines [21] and safety concerns over viral-vector delivery in hepatocyte maturation, Du and colleagues recently proposed a novel method using only a small molecule cocktail to drive cell fate transitions [22]. This cocktail includes small molecules like CHIR99021, dimethyl sulfoxide (DMSO), sodium butyrate, A83-01, and FPH1 [22] and resulted in DE induction and complete hepatic specification within 13 days, yielding functional cells with the capacity to produce albumin and store glycogen [22]. Growth factors, such as activin A, FGF, and hepatocyte growth factor (HGF), are known for their high cost and relatively lower stability versus small molecules [21]. Thus, more efficient stimulation of Nodal and Wnt signaling pathways for DE differentiation can be induced by stepwise application of CHIR99021, inducer of definitive endoderm 1 (IDE1), and PD0332991 [21,23].

#### 3.1.2. Hepatic Specification & Maturation

Liver development relies on intricate cellular and molecular processes involving endodermal patterning and signaling molecules for formation of hepatocytes and overall liver structure [13]. During the hepatic specification stage, key signaling pathways such as Wnt/β-catenin, TGF-β, and BMP play crucial roles in regulating the expression of transcription factors like hepatocyte nuclear factor 4 alpha (HNF4α) and GATA4, which are essential for hepatoblast formation. The Wnt/β-catenin pathway, in particular, is both necessary and sufficient for liver specification, with overexpression of Wnt2bb or Wnt8a inducing ectopic hepatoblast formation in zebrafish [13,24]. The TGF-β pathway, conversely, is involved in regulating cell proliferation and differentiation during liver development, with its inhibition promoting the expansion of hepatoblasts. The BMP pathway regulates the expression of transcription factors involved in liver development, and its activation induces HNF6 expression.

Immature hepatocyte formation is followed by their maturation [25]. HGF is crucial for the hepatic endoderm (HE) formation and subsequent liver development, inducing differentiation of hepatoblasts into hepatocytes [26,27]. Synergistic use of HGF and other growth factors, such as dexamethasone, FGF2, FGF4, BMP4, activin A, Wnt3a, and Oncostatin M (OSM), promotes hepatocyte differentiation [13,27,28,29]. Where HGF was used to induce differentiation, increased expression of endodermal markers, such as forkhead box protein A2 (FOXA2), along with Wnt3a and activin A, was observed compared to where HGF was absent (39.3% vs. 14.2%, respectively). HGF elicits a synergistic effect on other signaling molecules, like Wnt3a, activin A, OSM, and glucocorticoid hormones (e.g., dexamethasone) [26,30].

During maturation, signaling pathways such as Wnt/β-catenin and Notch establish the zonation pattern of hepatocytes, with Wnt signals from central-vein endothelial cells establishing the central hepatocyte zonation and Notch signals establishing periportal hepatocyte zonation. Additionally, modulation of signaling pathways such as FGF and HGF can enhance the functional properties of differentiated hepatocytes [31].

Differentiation of DE into hepatocytes, including the priming process, takes ~7 days, while hepatic maturation, with continued supplementation of HGF, Oncostatin M (OSM) and other hepatic morphogens, can take several weeks [32,33]. Xie and colleagues [32] reported hepatic maturation from iPSCs ranging from 9–25 days, depending upon cell expansion and culture conditions.

Immature hepatocytes are subsequently incubated in hepatocyte maturation and maintenance medium containing HGF and OSM, inducing expression of maturation transcription factors, such as HNF-4α and enhancer binding protein-alpha (EBPα) [13,33,34]. Cells obtain maturity and functional capacity with supplementation of reagents such as DMSO, insulin, glutamine, dexamethasone, and FGF4 [33,34]. Later in differentiation, characteristic cuboidal morphology, hepatocyte marker gene expression, and functional markers (glycogen storage, albumin production) are observed [35,36].

Hepatocytes that closely resembled primary human hepatocytes morphologically, with high hepatocyte protein marker expression (CYP450) and functionality were produced when DE was treated with DMSO, HGF, dexamethasone, OSM, and N-hexanoic-Try-Ile-(6)-amino hexanoic amide (dihexa) versus treatment with BMP4, FGF2, and insulin [23,35].

To better replicate liver structure and microenvironments, three-dimensional (3D) cultures, such as liver organoids, have been developed, utilizing extracellular matrix and microfluidic chips coated with collagen or Matrigel to mimic in vivo conditions such as shear stress [37,38]. Human iPSCs have been cultured on a liver-on-a-chip device made of poly(dimethylsiloxane) (PDMS) for the formation of embryoid bodies and, ultimately, liver organoids [37].

**Figure 2:**
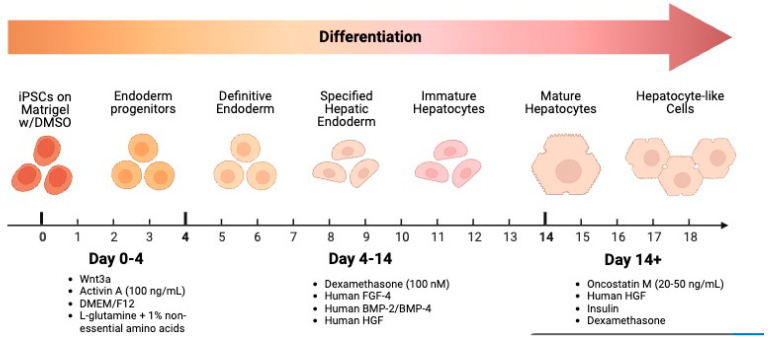
Differentiation of hiPSCs into human hepatocytes through different stages [13,28,34].

A general schematic of the generation of heaptocytes from iPSC is represented in Figure 2, and the published hepatocyte differentiation protocols are presented in Table 1.

### 3.2. Evaluation

Assessing iPSC-derived hepatocyte functionality using certain indicative markers is essential prior to applications such as transplantation or disease modeling [20]. Testing for functionality is typically performed 2–3 days following maturation due to limited stability [34].

In vivo liver functions, especially those related to insulin sensitivity, must be mimicked by iPSC-derived hepatocytes, including albumin production, glycogen storage, drug metabolism, and ammonia elimination; however, neonatal hepatocytes show reduced enzyme expression and thus have lower metabolic tolerance (e.g., for drug metabolism) [27,43]. Interestingly, hepatocyte maturation in a liver-like microenvironment has been shown to increase the expression of genes involved in insulin signaling and lipogenesis—key pathways required for insulin target cells [44].

Album production is assessed using enzyme-linked immunosorbent assay (ELISA), whereas glycogen storage is assessed using the periodic-acid Schiff (PAS) stain, which binds to glycogen within hepatocyte cytoplasm [45,46]. Indocyanine green (ICG) uptake and release can unambiguously confirm the functional presence of hepatic transporters. The drug metabolizing capacity of the hepatocyte-like cells (HLCs) is key for pharmaceutical applications, drug inducibility of CYP enzymes being indicative of drug metabolizing capacity. Studies investigating basal and drug-induced CYP activity in stem cell-derived HLCs reported CYP activity that was further stimulated by drugs [47].

Assessing drug metabolism using a range of rifampicin concentrations in iPSC-derived hepatocytes is heavily reliant on CYP3A4 expression, which is responsible for metabolizing > 50% of clinically approved drugs [48]. Assessment is performed by treating cultivated iPSC-derived human hepatocytes with a range of rifampicin concentrations (0, 5, 10, or 20 μM) and subsequently determining CYP3A4 expression [48]. Gene expression analyses and immunocytochemistry of key hepatocyte transporters, such as the bile-secreting ATP binding cassette (ABC) transporter, are also conducted [46].

Given the concurrent incidence of non-alcoholic fatty liver disease (NAFLD) with type 2 diabetes, determining lipid accumulation within iPSC-derived hepatocytes is necessary prior to their therapeutic use in diabetic patients [49,50]. Low-density lipoprotein and its receptor (LDL and LDLR, respectively) are both implicated in the pathogenesis of NAFLD; as such, the reduction of its central protein component, apolipoprotein B (ApoB), is investigated by ELISA to assess hepatocyte lipid-accumulating tendencies [50]. Reduction of ApoB reflects absent LDLR activity due to receptor internalization with inability to clear LDL, rendering the hepatocyte unsuitable for therapeutic use [50,51].

### 3.3. Application

A major use of hiPSC-derived hepatocytes is for modeling complex diseases, like obesity and NAFLD, that can be difficult to study in rodent models or human tissue [49]. iPSC-derived hepatocytes from normal and obese subjects are cultured to assess their lipid accumulation, via hepatic fibrosis pathways, by running transcriptome analyses to compare gene expression associated with hepatic fibrosis (caveolin 1 (CAV1) and cluster of differentiation 36 (CD36)) [49]. Mutant iPSC-derived hepatocyte models deficient in the transcription factor have been developed to determine the role of this protein in hepatocyte functionality and endoplasmic reticulum (ER) stress; loss of *FOXA2* resulted in upregulated lipid accumulation and ER stress, with reduction in albumin synthesis and glucose uptake [52].

A major etiological criterion of T2D, hepatic IR, can be studied in hiPSC-derived hepatocytes to explore hormonal effects on glucose metabolism and production by the liver. Groeger and colleagues investigated insulin sensitivity and macrophage-mediated inflammation in iPSC-derived hepatocytes following a 24 h starvation period deficient in insulin, glucocorticoids, and other growth factors [53]. Notably, IR in hepatocytes when co-cultured with macrophages was associated with cytokines tumor necrosis factor-alpha (TNFα) and interleukin-1 beta (IL-1β) [53].

The direct therapeutic use of iPSC-derived hepatocytes has been implemented for the treatment of several liver diseases [8] including familial hypercholesterolemia, glycogen storage diseases, and alpha-1 antitrypsin (A1AT) deficiency [54]. As such, iPSC-derived hepatocytes have shown potential in regenerative medicine and liver regeneration following transplantation [55] with reduction in liver fibrosis and rescue of fulminant liver failure in rodent models [26,56]. Further studies are needed for treatment of human liver disease, especially for T2D and its associated co-morbidities. Importantly, the close HLA match that iPSC-derived hepatocytes provide negates the need for immunosuppression [28], potentially transforming diabetes management.

## 4. iPSC-Derived Skeletal Muscle

### 4.1. Stages, Signaling Molecules, and Growth Factors

#### 4.1.1. iPSC Generation

Reprogramming of somatic cells into iPSCs prior to their differentiation into skeletal muscle parallels that of hepatocytes. Fibroblast-derived iPSCs are typically used for skeletal muscle differentiation; however, use of myoblast-derived iPSCs has recently been investigated given its heightened efficiency in differentiating into the myogenic progenitor, the satellite cell [57]. Owing to preservation of epigenetic myogenic memory, myoblasts reprogrammed into iPSCs demonstrate a higher yield of myocytes and regenerative satellite cells [58]. Transfection of myoblasts with *OCT4* suppresses myoblast determination protein 1 (MyoD1) expression, a critical gene in myogenic differentiation, which is necessary for the reprogramming of myoblasts to pluripotent precursors [57,59].

#### 4.1.2. Mesoderm Induction and Paraxial Mesoderm Formation

Mesoderm induction and paraxial mesoderm formation are critical stages in the differentiation of iPSCs into skeletal muscle regulated by key signaling pathways and in vivo morphogens, such as Noggin and BMP [60]. BMP signaling plays a role in mesoderm induction and patterning, its primary function being specification of ventral/lateral mesoderm fates [61]. Mediolateral axis patterning is driven by gradients of these morphogens, along with notochord and neural tube signaling, which drives differentiation into the paraxial mesoderm. Segmented regions of this paraxial mesoderm, termed somites, are exposed to different concentrations of signaling molecules, specifically retinoic acid (RA) and those from the Wnt and FGF (FGF2) families. Wnt signaling, in particular, is essential for mesoderm induction and patterning, with maternal Wnt signals promoting dorsal/medial mesoderm fates and zygotic Wnt signals promoting ventral/lateral mesoderm fates [61,62,63]. FGF signaling is also critical for the induction of posterior mesoderm and its patterning into dorsal/medial fates. The paraxial mesoderm, which gives rise to the somites and ultimately the skeletal muscles, is specified by the expression of transcription factors such as T-box transcription factor 6 (Tbx6) and mesoderm posterior BHLH transcription factor 1 (Mesp2.1). This exposure induces expression of key driver genes for mesodermal differentiation, such as *mesogenin 1 (MSGN1)* and *T-box transcription factor 6 (TBX6)* and, notably, key transcription factors for myogenic differentiation, *myogenic factor gene 5 (Myf5)*, and paired box genes 3 and 7 *(PAX3; PAX7)* [64].

In vitro overexpression of these transcription factors is induced via mRNA or non-integrative vectors [60,65], delivering a pure, rapid, and efficient yield of myogenic progenitors [66]. In serum-free medium cultures, iPSCs are typically first treated with BMP4 to initiate their differentiation into mesodermal cells, after which BMP is discontinued to prevent generation of lateral mesodermal cells, like osteocytes and cardiomyocytes [67]. Subsequent coupling of BMP inhibition, using molecules such as LDN193189, with Wnt pathway activation (Wnt3a) drives the mesodermal precursor toward a myogenic fate [59,68]. Paraxial mesoderm is first formed, manifested by the morphogen-induced expression of myogenic transcription factors (i.e., PAX3) [68]. The human iPSC-derived cells are then cultured in medium with growth factors (insulin-like growth factor 1 (IGF1) and HGF), after being sorted according to myogenic differentiation markers [59]. Other, less-common induction cocktails have also proved successful at inducing PAX3+ myogenic precursors and include factors like glycogen synthase kinase 3 beta (GSK3β) inhibitor, bFGF, and dexamethasone [69]. Within two weeks, paraxial mesoderm yields myoblasts, which further differentiate into myotubes before maturation into skeletal muscle cells [59].

#### 4.1.3. Myoblast Specification & Proliferation

Specification of myogenic progenitors from paraxial mesoderm is driven by activation of FGF, IGF, and HGF signaling pathways that promote the expression of transcription factors such as PAX3 and PAX7, essential for commitment of cells to the myogenic lineage [70]. Myogenic progenitor cell specification then ensues through expression of terminal myogenic differentiation markers: MyoD and Myf5 [71,72]. FGF signaling promotes myoblast proliferation through the activation of Wnt/β-catenin signaling [73]. Additionally, silent mating type information regulation 2 homolog 1 (Sirt1), a NAD+-dependent deacetylase, enhances myoblast cell line C2C12 myoblast proliferation by inhibiting the myostatin signaling pathway. Myostatin, a member of the TGF-β superfamily, is a negative regulator of skeletal muscle growth and inhibits myoblast proliferation [74]. The significance of the myoblast specification and proliferation stage is in the generation of progenitor cells that ultimately fuse to form mature skeletal muscle fibers, a key step for optimization of disease modeling, regenerative medicine, and drug screening [60].

iPSC-derived mesodermal cells cultured with a GSK3β inhibitor (CHIRO99021) and a BMP inhibitor (LDN193189) [68], followed by treatment with principal growth factors (HGF, IGF1, and FGF2) induces a myogenic response [75]. Myogenesis is typically detected after ~3 weeks in culture [68]. Benefit from addition of the Notch signaling modulator, (S)-tert-butyl 2-((S)-2-(2-(3,5-difluorophenyl)acetamido)propanamido)-2-phenylacetate molecule (DAPT), which mimics its in vivo counterpart, Delta1, aids conversion of myogenic precursors (PAX3+ and PAX7+) to differentiated myoblasts (MyoD+ and Myf5+) [59,76]. Given the demonstrated role of TGFβ and its agonists (growth differentiation factor 15 (GDF 15), myostatin) in inhibition of myogenic differentiation [77,78], daily administration of a TGFβ inhibitor, such as SB431542, is recommended [60,79].

#### 4.1.4. Myotube Formation & Maturation

Following myoblast proliferation and subsequent TGFβ inhibition, terminal differentiation of multinucleated myoblasts occurs, leading to cell fusion into multinucleated myotubes, as a result of the key transcription factor—myogenin (MyoG) [59]. In addition to myotubes, another primary component of skeletal muscle that myogenic progenitors give rise to is the regenerative satellite cell, characterized by expression of PAX7 [66]. Vandenburgh and colleagues investigated myotubes as building blocks for the formation of striated myobundles, whereby myotube alignment is facilitated by incorporating collagen and fibrin in hydrogel cultures [80]. Within culture, compaction is induced by both myotubes and fibroblasts that create alignment-producing tension, whereas induction of contraction and extracellular formation is dependent on force-generating fibroblasts. Various myotube alignment frame systems, which induce alignment of myotubes in the direction of stress, have been studied, including fixed end, microchannel, and deformable post systems [80].

Myogenic progenitor cultures are supplemented with DMEM containing low serum FBS, insulin, IGF-1, and OSM, which induce differentiation into myotubes within ~4 weeks [60,69,79]. Removal of doxycycline from the myogenic progenitor culture promotes a terminal myotube fate with MyoD expression [81].

Other differentiation factors typically incorporated into culture include temporary exposure to a BMP pathway inhibitor, like LDN193189, and a Notch pathway inhibitor, like DAPT, which induce muscle neuron and motor neuron differentiation, respectively [60]. Myoblast fusion into myotubes and corresponding cell twitching are observed after supplementation with myotube differentiation factors, confirmed by expression of MyoD, desmin, and myogenin [80].

Tissue engineering procedures using piezoelectrical materials, such as polyvinylidene fluoride (PVDF), improve in vitro myogenic differentiation through mechanically induced electric stimulation [82,83]. Myotube contractility is modulated and promoted by electrical stimulation strategies [84]. Magnetic field-induced stimulation also promotes differentiation of muscle cells into more functional myotubes, guided by use of magnetic nanoparticles (e.g., Fe_3_O_4_) and can induce significant myotube contraction [85].

Table 2 outlines protocols for iPSC-derived skeletal muscle suitable for transplantation into humans. Another strategy utilizes direct reprogramming of iPSCs into myogenic lineage using overexpression of transcription factors like PAX3, PAX7, and MyoD. This approach can bypass the intermediate mesoderm and progenitor stages; however, these transgene-based methods may not fully recapitulate the physiological differentiation process. Regardless of the specific protocol, differentiation of iPSCs into functional skeletal muscle cells remains a challenge, with variability in efficiency and maturity of the resulting cells. Ongoing research aims to optimize these protocols, improve the scalability and reproducibility of the differentiation process, and explore the use of 3D culture systems and biophysical stimuli to enhance maturation of iPSC-derived skeletal muscle [60].

### 4.2. Evaluation

At each differentiation stage, myogenic cells are assessed for their expression of cell stage-specific markers. Gene expression analyses are conducted at each stage using a real-time polymerase chain reaction (RT-PCR) [92]. Expression of key structural and functional proteins, such as myosin heavy chain, troponin, and acetylcholine receptors, is commonly assessed using immunocytochemistry, Western blotting, and gene expression analysis [70,91].

Terminal specification of myoblasts is primarily evaluated by expression of MyoD1 and Myf5; MyoG expression is used to assess myoblast fusion into multinucleated myotubes and, ultimately, terminal skeletal muscle cell differentiation. Immunocytochemistry is used to characterize expression of key protein markers and myofibers within skeletal muscle cells, such as lamin A/C and human spectrin (sarcolemma) [70,92].

To evaluate contractile properties, techniques like video microscopy, atomic force microscopy, and electrical field stimulation have been used to analyze the contractile kinetics, amplitude, and frequency of iPSC-derived myotubes [93,94].

Measurement of plantar flexion following myogenic progenitor cell injection, albeit in a mouse model [70], and other tools, such as the myotube analyzer investigated by Noë and colleagues, aid in the assessment of muscle function following differentiation [95].

Regarding insulin responsive functionality, investigations into glucose transporter type 4 (GLUT4) expression, myofiber structure, and mitochondria function (via a mitochondrial membrane potential assay) must be performed [76]. Western blotting analysis can be used to measure phosphorylation of the insulin receptor (INSR), insulin receptor substrate-1 (IRS-1) and extracellular-signal-regulated kinases (ERK1/2) in iPSC-derived myotubes, thereby reflecting insulin responsiveness within these cells and their functionality for treatment of IR in T2D [96].

Electrophysiological measurements, including assessment of action potentials and ion channel activities, can determine excitability and functional integration of iPSC-derived skeletal muscle cells, patch-clamp recordings and multi-electrode array (MEA) systems being employed for this purpose [60]. Recently, functional contractility of iPSC-derived skeletal muscle fibers has also been effectively assessed by measuring intracellular calcium concentrations (using calcium-dependent fluorescence changes) following electrical field stimulation [97]. Metabolic assays, such as glucose uptake, lactate production, and mitochondrial respiration, can be used to evaluate the energy metabolism of the cells.

The ability of iPSC-derived skeletal muscle cells to respond to various stimuli, such as electrical, mechanical, or pharmacological cues, can provide valuable information about their maturity; the use of bioreactors and engineered muscle tissues can facilitate assessment [98,99].

### 4.3. Application

iPSC-derived skeletal muscles and myotubes have potential applications in research and therapy. These cells can model key pathological features of IR, including reduced glucose uptake, mitochondrial dysfunction, and abnormal gene expression offering valuable insights into disease mechanisms and the development of personalized treatments. iPSCs generated from individuals with IR, such as T2D, can be differentiated into skeletal muscle cells and can have therapeutic applications in restoring insulin-responsive muscle mass [68,100,101]. With respect to iPSC-derived skeletal muscle cell use in the setting of diabetes, Wang and colleagues reported that an increased rate of myoblast serial passaging decreases their proliferation rate, increases DNA damage, and reduces their sensitivity to insulin [77] with reduced insulin responsiveness to glucose uptake, glycogen synthesis, and lipid metabolism. Given the limited proliferation capacity of myotubes, their suitability for transplantation is suboptimal [59]. Patient-specific iPSC-derived myotubes provide a physiologically relevant platform for high-throughput drug screening to improve insulin sensitivity [102]. Functional readouts, including contractile and metabolic activity, enable evaluation of drug efficacy.

Muscle repair and regeneration comprise the mainstay of iPSC-derived skeletal muscle applications for musculoskeletal disorders like Duchene muscular dystrophy (DMD), Miyoshi myopathy, Becker muscular dystrophy (BMD), and, more recently, IR in T2D [103,104]. Obesity, hyperinsulinemia, and IR were detected in patients with Duchenne muscular dystrophy (DMD) and Becker muscular dystrophy (BMD), regardless of corticosteroid treatment. While transplantation and xenografting have been successfully carried out in rodent models, there is still a need to explore the potential of patient-specific iPSC transplantation of skeletal muscle cells, myogenic progenitors, and satellite cells for the treatment of human diseases.

Some challenges may be present upon transplantation of iPSC-derived tissue, such as early cell death and limited cell proliferation; therefore, the regenerative capacity of cells prior to transplantation must be adequate [105].

Myogenic precursor induction factors, particularly GSK3β inhibitors, show benefits in T2D by modulating insulin secretion during chronic hyperglycemia [106]. Phosphoproteomic studies reveal GSK3β’s role in T2D pathogenesis and beta cell failure, highlighting its therapeutic potential. Additionally, Batista et al. identified multiple insulin signaling defects in myocytes from T2D iPSCs, including issues with Rho-GTPase regulation, vesicular trafficking, and nuclear processes [107]. Thus, differentiating iPSCs into skeletal muscle with GSK3β inhibitors provides a valuable model for studying T2D cellular mechanisms.

One of the main shortcomings observed in skeletal muscles affected by T2D is their diminished glucose uptake due to IR [108]. iPSC-derived myotubes from T2D patients retained those IR properties [108]. Currently, there is limited available data regarding the effective utilization of skeletal muscle tissue and satellite cells in diabetic animal models versus animal models of other muscle myopathies or in diabetic patients. Despite the therapeutic potential of this approach, further research is needed.

## 5. iPSC-Derived Adipocytes

Adipocytes are the specialized cells that store energy as fat [109]. Brown (BAT), white (WAT), and beige adipocytes are the three different types of adipocytes in humans, and they differ significantly in both structure and function. Brown adipocytes generate heat through non-shivering thermogenesis, while white adipocytes store excess energy as triglycerides, and beige adipocytes exhibit characteristics of both and contribute to energy expenditure. Their presence and functionality impact insulin sensitivity, lipid metabolism, and inflammation. BAT is associated more with subcutaneous rather than visceral adipose tissue, resulting in decreased central obesity [110]. Studying these adipocytes is crucial for understanding energy balance regulation and conditions such as obesity and metabolic syndrome. Moreover, therapeutic interventions targeting adipocyte populations hold promise for improving metabolic health.

The generation of white, brown, and beige adipocytes from iPSCs has allowed for deeper insights into adipose tissue biology and potential therapeutic innovations. Key signaling molecules and pathways are instrumental in directing the differentiation of these distinct adipocyte types.

### 5.1. Stages, Signaling Molecules, and Growth Factors

Adipogenesis occurs in two phases: commitment (derivation of pre-adipocytes from mesenchymal stem cells) and terminal differentiation (development of pre-adipocytes into mature fat cells) [111]. Mesenchymal stem cells (MSCs) possess the ability to generate both white and brown adipocytes. White and brown adipocytes within WAT share a Myf5− progenitor, while brown adipocytes in BAT originate from a Myf5+ progenitor with both myogenic and adipogenic potential [112]. Adipogenesis is regulated by various signaling pathways and factors, including IGF-1, glucocorticoids, cAMP, BMP2/4/7, ERK/MAPK, p38/MAPK, and Ras. Epigenetic mechanisms also contribute, involving chromatin remodelers, histone modifiers, and epigenomic readers [111,113].

#### 5.1.1. Derivation of Mesenchymal Stem Cells (MSCs)

Taura et al. [114] first showed that iPSC cells have adipogenic potential comparable to human ES cells. This study leveraged embryoid body (EB) formation to differentiate pluripotent stem cells into the three germ lineages using Dulbecco’s modified eagle medium (DMEM/F12) supplemented with 20% knockout serum replacement (KSR) with transient RA treatment from day 3 to day 5 [115]. After 12 days in suspension, EBs were transferred to fibronectin/poly-L-ornithine-coated plates. Ten days post-plating, adherent outgrowths appeared and were maintained for a week in mesenchymal growth medium (DMEM + 10% FCS + 5 ng/mL FGF2). The outgrowth cells were cultured until a homogeneous fibroblastic morphology appeared, which could then further differentiate into adipocytes [116,117].

#### 5.1.2. Adipocyte Differentiation from MSCs

Adipocyte differentiation can be induced using an adipogenic cocktail or transduction of adipogenic factors. Taura et al. used a cocktail of adipogenic factors including insulin, 3-Isobutyl-1-methylxanthine (IBMX), dexamethasone, indomethacin, and pioglitazone, to induce adipocyte differentiation [114]. IGF-1 signaling induces pre-adipocyte differentiation in vitro; pre-adipocytes have many IGF-1 receptors to which insulin binds at non-physiologically high concentrations [111]. IGF-1, regulated by growth hormone, stimulates pre-adipocyte differentiation via MAPK signaling and can counteract Pref-1-mediated inhibition. Insulin and glucocorticoids, such as dexamethasone, enhance adipogenesis and insulin sensitivity [111,118,119]. Indomethacin at high concentrations can induce adipogenic differentiation of preadipocytes by promoting expression of peroxisome proliferator-activated receptor gamma (PPARγ; the master regulator of adipogenesis) [120].

In mammals, PPARγ exists in two isoforms: γ1 (G1) and γ2 (G2). MSCs expressing PPARγ2 differentiate into white adipocytes (WACs), while those expressing PPARγ2 along with C/EBPβ and PR domain-containing 16 (PRDM16) differentiate into brown adipocytes (BACs). Meanwhile, 3-Isobutyl-1-methylxanthine (IBMX), a competitive, nonselective phosphodiesterase inhibitor, increases intracellular cAMP levels and activates protein kinase A (PKA), enhancing transcription of PPARγ and expression of adipogenic genes. Additionally, dexamethasone and IBMX both act as inducers of C/EBPδ and C/EBPβ [121]. Pioglitazone, a PPARγ activator, also promotes adipogenesis.

Another method of generating adipocytes from mesenchymal progenitor cells (MPCs) is transduction with doxycycline-induced *PPARG2* (~88% success). These adipocytes displayed an unilocular morphology and expressed markers such as CCAAT-enhancer binding protein alpha (CEBPA), fatty acid binding protein 4 (FABP4), and hormone-sensitive lipase (HSL), indicative of WAT formation. To generate BATs, MPCs transduced with constructs containing *PPARγ2*, *CEBPB*, *and PRDM16* were cultured in doxycycline-containing adipogenic media; after 21 days, the adipocytes exhibited a multilocular lipid droplet morphology, abundant mitochondria, and strong cytoplasmic uncoupling protein 1 (UCP1) staining, characteristic of BATs. Another method of adipocyte generation using EBs and culturing outgrowths on adherent plates in the presence of hematopoietic factors such as BMP4 and BMP7, produced cells with extensive multilocular lipid droplet formation; the cells showed 95% positivity for UCP1 and were rich in mitochondria with transverse cristae [122,123,124].

TGF-β exerts a primarily inhibitory influence on adipogenesis via suppressor of mothers against decapentaplegic (SMAD3) signaling by suppressing expression of C/EBPs and PPARγ and inducing PPARγ phosphorylation, thus inhibiting the adipocyte commitment of bone marrow-derived mesenchymal stem cells [125,126]. Similar to TGF-β, activin A primarily exerts an inhibitory effect on adipogenesis, enhancing the proliferation of human adipocyte precursors while suppressing their differentiation [126,127].

#### 5.1.3. Protocol

**Figure 3:**
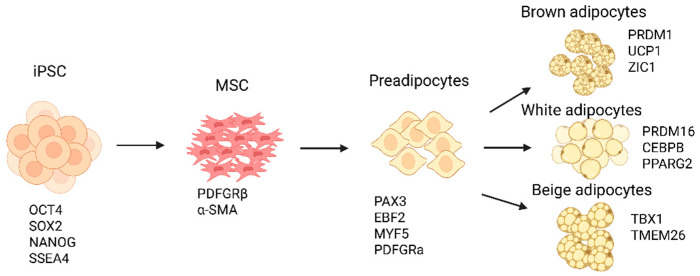
Differentiation of hiPSCs into human adipocytes (brown, white, and beige) and the markers used to evaluate cells obtained at the end of each stage.

For iPSCs to produce adipocytes, a stepwise process occurs which can be divided into three main stages: hiPSCs to MSCs to adipocytes. The process of hiPSCs differentiating into MSCs ensures the purification and scalability of the stem cells, which are crucial for differentiation into BACs or WACs [128,129]. hiPSC-derived adipocytes hold significant promise for transformative medical advancements; however, the protocols required for their derivation are highly complex and labor-intensive; see Table 3 [130]. A general schematic representing the generation of different adipocytes from iPSC is shown in Figure 3.

### 5.2. Evaluation

The generation of white, brown, and beige adipocytes from iPSCs holds promise for research and therapeutic applications. To ensure the reliability and reproducibility of these iPSC-derived adipocytes, thorough evaluation, characterization, and validation are essential. Here, we review key aspects of this process.

Adipose tissue releases adipokines, inflammatory factors, and free fatty acids [137,138] and has a primary function of glucose metabolism. Radioactively labeled glucose can be measured in vitro via adipose tissue needle biopsies from abdominal and femoral regions after ~4 h of glucose administration [139]. To ensure reliability, radioactively labeled triglycerides must be measured in repeated biopsies at day 1, week 1, and monthly up to 7 months following glucose administration [139]. Glucose uptake can also be measured by quantifying glucose transport proteins such as GLUT4; GLUT4 is compartmentalized within intracellular vesicles and, upon glucose administration, GLUT4-containing vesicles fuse with the plasma membrane, affecting an increase in intracellular glucose concentration [140]. By using enhanced green fluorescent protein (EGFP) green fluorescence to stain GLUT4 plus flow cytometry, the total number of reporter GLUT4 proteins can be quantified [141].

Mature adipocytes do not proliferate, and viability is determined using trypan blue staining (though this can be unreliable, as nonviable adipocytes may be counted) [142]. Lee and colleagues developed a method using a viability dye to identify viable adipocytes that can be manually quantified [143].

Glucose-6-phosphate dehydrogenase (G6PD), a rate-limiting enzyme of the pentose phosphate pathway (PPP), has been implicated in tissue inflammation and systemic IR in obesity [144]. G6PD deficiency improves IR with reduced adipose tissue inflammation in obesity; thus, G6PD levels can be used as a marker of adipocyte activity [145]. Adipocyte differentiation is associated with an increase in intracellular lipid droplets. These lipid droplets are highly organized organelles, consisting of a neutral lipid (triglycerides and cholesterol esters) core and an outer phospholipid layer with various embedded regulatory proteins. At the cellular level, adipocyte differentiation is commonly visualized microscopically following staining with lipid-specific stains: Oil Red O, Nile Red (NR), and Bodipy 493/503 (BDP). Characterization of the gene expression profile of mature adipocytes includes markers such as *leptin*, *aP2 gene product protein (aP2)*, *PPARγ2*, *UCP-1*, and glucose transporters assessed by quantitative reverse transcription polymerase chain reaction (qRT-PCR), and transcription factors *peroxisome proliferator-activated receptor-γ coactivator (PGC1A)* and *PRDM16* characterize beige and brown adipocytes. Extracellularly released metabolite and adipokine profiling (glycerol, free fatty acids (FFAs), adiponectin, and leptin) can be measured using ELISA. WACs can be identified by their large unilocular lipid droplets and expression of white-specific markers like leptin and resistin [146]. BACs are characterized by multilocular lipid droplets, high mitochondrial content, and expression of UCP1, PGC1α, and PRDM16 [146,147]. Beige adipocytes display a mixed phenotype, with moderate lipid droplet size, UCP1 expression, and upregulation of beige-selective markers like cbp/p300-interacting transactivator 1 (CITED1), CD137, and transmembrane protein 26 (TMEM26) [146]. Lipolysis can be measured by quantifying glycerol and free fatty acid release in response to adrenergic stimulation. Functional assays, such as glucose uptake and lipolysis, should yield similar results to those of primary adipocytes [136].

The key metabolic feature of brown and beige adipocytes is their capacity for uncoupled respiration where proton leakage through the UCP1 conduction channel drives heat production at the expense of ATP synthesis. To investigate the metabolic activity of these cells, extracellular acidification rates (ECARs) and oxygen consumption rates (OCRs) can be determined using a Seahorse Bioanalyzer. These assays determine glycolytic flux and rates of oxidative phosphorylation, respectively. The role of interleukin 4/13 (IL-4/13) pathway in the body’s response to cold exposure was demonstrated by exposure of WT and Il4/13^−/−^ mice to progressively colder temperatures, where increased oxygen consumption in WT mice, a response that was blunted in Il4/13^−/−^ mice, was observed, especially at 4 °C [148]. To validate the physiological relevance of iPSC-derived adipocytes, comparison to native adipocytes is recommended, and to confirm iPSC-derived adipocyte identity, gene expression profiles can be compared to primary adipocytes using RNA sequencing [149]. Seahorse XF metabolic analysis can determine whether the molecular profile of iPSC-derived adipocytes demonstrates enhanced metabolic activity.

### 5.3. Application

IPSC-derived adipocytes provide a valuable tool for mechanistic studies of IR and metabolic dysfunction, drug testing, as well as adipocyte development and cell-based transplantation therapies. iPSC-derived brown and beige adipocytes can be used to investigate their roles in modulating energy expenditure and glucose metabolism, potentially uncovering therapeutic targets for IR.

Familial partial lipodystrophy type 2 (FPLD2) is an autosomal dominant genetic disorder that, if left untreated, can result in insulin-resistant diabetes mellitus [118]. It is characterized by severe defects in adipocyte function and adipogenesis. Adipocytes in FPLD2 exhibit markers of autophagy and increased expression of proteins involved in triglyceride catabolism. In this case, adipose tissue transplantation is effective and, additionally, is antidiabetic, though a large adipose tissue transplant is needed to reverse diabetes [150].

Transplantation of both WAT and BAT produces endocrinological benefits such as regulation of glucose homeostasis and improved insulin sensitivity [151]. Humans with lipodystrophies demonstrate downregulation of circulating exosomal miRNAs that is normalized by white and especially brown adipose tissue transplantation (adipose tissue contains a dense reservoir of circulating exosomal miRNAs). These miRNAs act as adipokines, regulating gene expression in adjacent tissues, thus improving glucose tolerance and reducing hepatic FGF21 mRNA and circulating FGF21 [152].

Adipocyte or adipose tissue transplantation has become widely used in reconstructive surgeries such as facial recontouring, hand rejuvenation, and body contouring [153,154]. Lipostructure is a long-lasting method of facial recontouring using autologous tissue, useful for cosmetic as well as medical causes, such as fractures and previous surgeries [153].

Subcutaneous transplants of embryonic BAT can correct T1D in streptozotocin-treated mice (both immune competent and immune deficient) with severely impaired glucose tolerance and significant loss of adipose tissue [155]. The process of transBATation provides resistance against diet-induced obesity by increasing overall sympathetic activity in the body [156].

However, several challenges remain in the application of iPSC-derived adipocytes for insulin resistance research and clinical therapy. Standardization of differentiation protocols to generate functional and mature adipocytes is necessary as well as development of efficient methods for large-scale production and purification.

## 6. iPSC-Derived Cardiomyocytes

iPSC-derived cardiomyocytes are valuable for cardiovascular research, modeling insulin resistance (IR), and exploring cardiac regenerative therapies. IR, a significant risk factor for heart disease, can be studied using iPSC-cardiomyocytes from IR patients, which show impaired glucose uptake, mitochondrial dysfunction, and altered gene expression [157,158].

### 6.1. Stages and Signaling Pathways

Cardiomyocytes are differentiated from iPSCs by recapitulating the pathway of in vivo differentiation, which includes the activin/Nodal, TGFβ, GSK3, Wnt, BMP, FGF2, and vascular endothelial growth factor (VEGF) signaling pathways. Activin A and BMP are crucial for the differentiation of iPSCs into cardiac mesoderm [159]. In cardiomyocyte proliferation, the assembly/activation of the TGF-β receptor complex occurs by binding of TGF-β ligand, which phosphorylates SMADs2/3 (canonical signaling) and epithelial transforming growth factor β-activated kinase 1 (TAK1; non-canonical signaling) [160]. Growth factors like insulin activate the PI3K/Akt pathway sequentially; GSK-3 is then inhibited and modulates the Wnt signaling pathway [161]. Differentiation stages from iPSCs to cardiomyocytes are induction, mesoderm specification, cardiac specification/expansion, and maturation [162,163].

#### 6.1.1. Mesendoderm Induction

The first stage involves the induction of mesendoderm, a precursor to both mesoderm and endoderm lineages. Activin A and BMP4, which activate the TGF-β and BMP signaling pathway, are commonly used to induce the expression of mesendoderm markers such as Brachyury (BRY) and Mixl1 [159].

#### 6.1.2. Cardiac Mesoderm Specification

The stage following induction is the specification of mesoderm into cardiac mesoderm, which involves inactivation of the Wnt pathway, inhibition of TGFβ, inhibition of p38 MAPK, inhibition of BMP, and activation of the smoothened receptor (SMO). The mesendoderm progenitors are then further specified towards a cardiac mesoderm fate through activation of Wnt signaling, along with FGF signaling, which promotes expression of cardiac transcription factors such as Mesp1 and NK2 homeobox 5 (Nkx2.5) [164].

#### 6.1.3. Cardiac Progenitor Commitment

The third stage is cardiac expansion and specification. Growth factors that develop this phase are VEGF, FGF2, and IGF-1 [163]. Other small molecules are involved, such as smoothened agonist (SAG), that help maintain cardiovascular progenitor cells. RA drives formation of pacemaker-like cells, and PD173074 enriches these cells. AG1478 inhibits epidermal growth factor receptor (EGFR) signaling, thus increasing nodal-like cardiomyocytes. Meanwhile, 1-Ethyl-2-benzimidazolinone (1-EBIO) activates SK channels (small-conductance Ca2+-activated K+ channels), causes selective survival of a subtype of cardiomyocytes, and depletes other cells types including ventricular-like cells [162,163,165,166].

As cardiac progenitors mature, they begin to express cardiac-specific genes and proteins such as T-box transcription factor 5 (Tbx5), Gata4, and myocyte-specific enhancer factor 2C (Mef2c) [167].

#### 6.1.4. Cardiomyocyte Maturation

The final stage involves terminal differentiation of cardiac progenitors into mature cardiomyocytes. This process is driven by the downregulation of Wnt signaling and the upregulation of calcium signaling pathways. Calcium-dependent transcription factors such as nuclear factor of activated T cells (NFAT) and calcium/calmodulin-dependent protein kinase II (CaMKII) promote the expression of contractile proteins and ion channels, leading to the formation of functional cardiomyocytes [168].

WY-14643, a PPARα agonist, and triiodothyronine hormone promote the maturation of cardiomyocytes [163]. The role of thyroid hormone in maturation is unclear; however, triiodothyronine increases the resting membrane potential, thereby supporting cellular excitability and contractility. One study found that triiodothyronine enhances cardiomyocyte differentiation, cardiogenesis, myofibrillogenesis, and the expression of calcium-handling proteins, resulting in more mature cardiomyocytes. Fatty acid treatment promotes cardiomyocyte hypertrophy, resulting in greater force production, improved calcium transients, increased action potential velocity, enhanced membrane capacitance, and enhanced mitochondrial function, all of which contribute to cardiomyocyte maturity [169,170,171]. Recent studies show that adding secreted frizzled-related protein 2 (sfrp2) improves sarcomere development and yields more mature cells than traditional protocols using pharmacological inhibitors [172].

#### 6.1.5. Protocol

**Figure 4:**
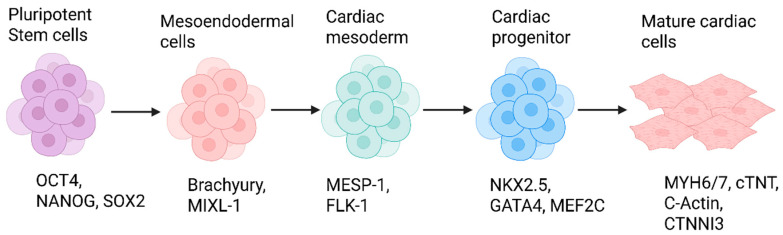
Differentiation of hiPSCs into mature cardiocytes and the markers used to evaluate cells obtained at the end of each stage.

In the laboratory, different procedures are used for differentiation, such as monolayer culture, embryoid bodies, and an inductive co-culture method [173,174,175]. Monolayer culture is further classified into direct differentiation and mixed sandwich. Embryoid bodies are classified into spin embryoid bodies, micropatterned embryoid bodies, and microwell embryoid bodies [162]. The monolayer culture method is currently the most efficient differentiation technique as it is simpler, more consistent, and yields more cardiomyocytes (>85%) compared to the other methods [175]. Cardiomyocyte differentiation protocols are presented in Table 4. A general schematic of representing the different stages of cardiomyocyte generation from iPSC is shown in Figure 4.

### 6.2. Evaluation

The stages of cardiomyocyte differentiation can be evaluated by examining the specific genes/cell markers that must be expressed at the end of each stage, either at the gene or protein level. Markers of mesodermal formation, BRY and Mixl1, are expressed during the induction stage. The genes that control the cardiogenic mesoderm proliferation stage are *MESP1*, *insulin gene enhancer protein (ISL1)*, and *kinase insert domain receptor (KDR)*. The cardiac progenitor cells differentiate into immature cardiomyocytes during the cardiac specification stage and the genes that control this process are *NKX2.5*, *GATA4*, *TBX5*, *MEF2C*, and *heart and neural crest derivatives-expressed protein 1/2 (HAND1/2).* Genes that direct final structural differentiation and sarcomere proteins are *myosin regulatory light chain 2 (MYL2)*, *MYL7*, *myosin heavy chain 6 (MYH6)*, and *troponin T2*, *cardiac type (TNNT2)* [180]. The biomarkers for the matured cardiomyocyte are MYL2, MYH7, and troponin I3, cardiac type (TNNI3) [163]. Structural phenotyping is conducted on cell attachment, and sarcomere packing density and orientation order parameters are analysed by computational analysis [181].

Human iPSC-derived cardiomyocytes display mixed atrial-, nodal-, or ventricular-like action potentials, but classification is complicated by culture conditions and immaturity. Patch clamp studies show their action potentials match normal QT intervals and respond to drugs as expected [182].

### 6.3. Application

Cardiovascular diseases are the primary cause of mortality in the world. iPSC-derived cardiomyocytes can be used as disease modelling tools for cardiac diseases such as diabetic cardiomyopathy and heart failure to better understand disease pathogenesis and for development of targeted treatments such as T2D-related cardiac dysfunction [183,184]. Implantation of these tissues, which can be engineered to secrete insulin-sensitizing factors, could help restore cardiac function and improve clinical outcomes in patients with IR and diabetes [185]. A recent study by Granéli et al. reported that IR can be induced in cardiomyocytes by lipid overload to establish a model that is close to diabetic cardiomyopathy [184].

iPSC-derived cardiomyocytes have been used for drug screening, disease monitoring, and regenerative and transplant medicine [186,187]. They are valuable for treating diseases such as congenital heart disease, drug-induced cardiac toxicity, inherited cardiomyopathies, and channelopathies [180,188]. Since 2011, they have been used in toxicology studies for drug and chemical safety testing and for high-throughput screening of drugs and compounds that could improve insulin sensitivity and cardiac function [189]. Contractile, metabolic, and signaling readouts can be used to evaluate candidate drug efficacy with the potential to accelerate development of novel therapies for IR-related heart disease [190].

Drawnel et al. utilized iPSC-derived cardiomyocytes to demonstrate that IR leads to increased oxidative stress and apoptosis through the p38 MAPK pathway, and treatment with a p38 inhibitor rescued these defects, suggesting a potential therapeutic approach [191]. Sharma et al. found that IR impairs calcium handling in iPSC-derived cardiomyocytes, leading to contractile dysfunction and suggesting calcium regulatory proteins as a therapeutic target [157].

iPSC-derived cardiomyocytes, in comparison to immortalized cell lines, are better for developing and appraising drug therapies [192]. Studies have used them to assess beta-blocker responsiveness (e.g., sotalol) and investigated dilated cardiomyopathy, revealing disrupted AMPK-sarcomere interactions and improved function with AMPK activators [193,194].

## 7. iPSC-Derived Neuronal Cells

### 7.1. Stages and Signaling Pathways

There are two major cell types in the brain: neuronal and glial cells. Both of these are further subclassified into various types, including glutamatergic neurons, dopaminergic neurons, GABAergic neurons, cortical neurons, astrocytes, oligodendrocytes, and microglia. Differentiation into a specific brain region requires targeted pathway induction, such as RA, Wnt/β-catenin, TGF/BMP, Notch, FGF, cytokines, Hedgehog, and C-Jun N-terminal kinase/Mitogen-activated protein kinase (JNK/MAPK) pathways [195,196].

The differentiation of iPSCs into neuronal cells involves a stepwise process regulated by various signaling pathways and transcription factors.

#### 7.1.1. Neuroectoderm Specification

iPSCs first differentiate into neuroectoderm. iPSCs differentiate into neuroectoderm through dual-SMAD inhibition using molecules like SB431542 and Noggin to block TGF-β and BMP pathways. RA and Wnt activation further guide neural induction, with early markers such as SOX1 and PAX6 indicating a successful transition [197]. Activation of the Wnt pathway is critical for neural induction and anterior–posterior patterning during early development. Use of dual-SMAD inhibition combined with 3D culture promotes efficient neuroectodermal induction [197,198].

#### 7.1.2. Neural Progenitor Specification

Neuroectoderm cells further differentiate into neural progenitor cells (NPCs), which are multipotent and give rise to neurons, astrocytes, and oligodendrocytes. The neural progenitors are then further specified towards a neuronal lineage. Active Notch signaling maintains NPCs in a proliferative state, helping to sustain the progenitor pool and delaying their differentiation. Simultaneously, the fibroblast growth factor (FGF)/ERK pathway plays a crucial role in maintaining NPC proliferation and preventing premature differentiation into neurons. Inhibiting Notch (e.g., using DAPT) induces expression of neuronal transcription factors like NEUROG1, NEUROG2, and ASCL1 [199,200]. Sonic hedgehog (Shh) signaling directs ventral patterning and subtype formation, such as motor neurons [201]. Forebrain-specific NPCs have been generated using Shh and Wnt modulators for modeling neurodevelopment and disease [202].

#### 7.1.3. Neuronal Commitment

As the neuronal progenitors mature, they express neuronal-specific genes and proteins. This stage is marked by activation of calcium signaling pathways and upregulation of key transcription factors such as NEUROG1 and NEUROG2, which regulate neuronal differentiation and maturation. NEUROG2 accelerates excitatory neuron formation [203]. Shh and Wnt pathways guide the development of specific neuronal subtypes—Shh for motor neurons, and Wnt for dopaminergic neurons. BDNF (brain-derived neurotrophic factor) and GDNF (glial cell line-derived neurotrophic factor) promote the survival and maturation of neurons. Neuronal progenitors in the form of neurospheres can be derived from iPS cells through Noggin treatment, PA6 coculture, or direct neural induction on laminin, resulting in early neural progenitors marked by Pax6, Sox1, and Sox2, which can subsequently differentiate into neurons, glia, and neural crest cells [204]. Neural crest progenitors can be obtained from hPSCs using CHIR99021 and BMP-2 [205].

#### 7.1.4. Neuronal Maturation

Terminal differentiation of neuronal progenitors into mature neurons involves the activation of synaptic signaling pathways, such as glutamate and gamma-aminobutyric acid (GABA) signaling [195]. Single-cell analysis of cerebral corticospheroids (hCSs) showed that astrocyte maturation progressing from radial glia to mature astrocytes was regulated by intrinsic and extrinsic signals. These pathways promote the expression of synaptic proteins and the formation of functional neuronal networks. Throughout these stages, various signaling molecules and pathways work in concert to guide the differentiation of iPSCs into neuronal cells [206]. hiPSC-derived cortical neurons cultured without glial support formed functional synaptic circuits with long-term potentiation [207]. Also, cholinergic neuron maturation can occur in adherent cultures with BDNF, GDNF, and laminin while dopaminergic neurons require BDNF, GDNF, and TGFβ3 [208,209].

#### 7.1.5. Protocols

iPSCs offer a versatile platform for studying neural development and neurodegenerative diseases, as they can be differentiated into various neural cell types. Numerous protocols have been developed to direct iPSC differentiation into specific neural lineages, each tailored to achieve distinct outcomes based on research objectives and target cell types. Published neural differentiation protocols are shown in Table 5.

### 7.2. Evaluation

Evaluation methods depend upon the type of neuronal cell being differentiated and the protocol followed. Evaluation of motor neurons is by immunohistochemistry staining, electrophysiological analysis of neuronal network connectivity, and transcriptome analysis by RT-PCR. Motor neuron markers, such as MN homeobox protein (HB9, transcription factor) and choline acetyltransferase (CHAT), are evaluated. In another study, cells were positive for astrocyte markers (GFAP, S100β, VIM, AQU4, ACSBG1, and APOE) after 30 days of culturing and were evaluated [214,215].

Kang and colleagues differentiated iPSCs towards both neural and glial progenitors and evaluated using a neuronal marker, class III β-tubulin (TUJ-1), and an astrocyte marker, glial fibrillary acidic protein (GFAP); both markers were positive in the neuronal progenitor cells [216,217]. The functionality of the iPSC-astrocytes can be confirmed by their response to inflammatory stimuli, as astrocytes release cytokines. Phagocytic functioning of microglia, calcium signaling, and uptake of glutamate can also be determined [218,219].

Nerve support markers and neurotrophic factors including human epidermal growth factor receptor 3 (ERBB3), GDNF, nerve growth factor (NGF), BDNF, and growth-associated protein 43 (GAP43) were also enriched in mature Schwann cells (SCs). Early SCs were enriched for early region 2 binding factor (E2F)— E2F7 and E2F8—whereas mature SCs expressed SRY box transcription factor 10 (SOX10) and Forkhead box protein O1 (FOXO1), POU domain class 3 transcription factor 2 (POU3F2), and T-box transcription factor 199 (TBX19) at higher levels. POU6F2 was the common enriched transcription factor in SC progenitor derivatives (SCPDs). Detailed assessment of SC cultures from hPSCs is performed by comparing their gene expression profile with primary SCs using the Single Cell Net machine, which classifies query scRNA-seq data and compares with reference datasets [220].

Whole-cell patch clamping is used to show iPSC-derived neuron ability to fire a series of action potentials [221]. Whole-cell patch clamp recording indicated that human iPSC-generated ChAT+ spinal motoneurons expressed large inward currents and outward currents by 8 weeks [222]. Co-cultures of iPSC-derived motor neurons with C2C12 muscle-like cells form myotube-like structures and connections of neurites with the myotubes. Aggregated bungarotoxin (BTX) staining is used to label postsynaptic acetylcholine receptors, located near synapsin, which is present in iPSC-derived motor neurons. This can be confirmed by confocal analysis [222]. To analyze the mixture of cell types that the cerebral organoids (Cos) were composed of, scRNA-seq analysis is performed.

### 7.3. Applications

iPSCs derived from individuals with IR disorders, such as T2D, can be differentiated into various neuronal subtypes, including sensory, motor, and autonomic neurons. These patient-specific iPSC-derived neurons can recapitulate key features of IR, such as impaired glucose uptake, mitochondrial dysfunction, and altered gene expression profiles, allowing for investigation of the underlying mechanisms by which IR affects neuronal function and survival.

Diabetes-induced neuropathy is a common complication that can lead to sensory, motor, and autonomic dysfunction. iPSC-derived neurons from diabetic patients have been used to model the pathological changes associated with diabetic neuropathy, revealing dysregulation of calcium homeostasis, oxidative stress, and upregulation of neuroinflammatory pathways. Several studies have shown their effectiveness in enhancing nerve function and alleviating peripheral neuropathy symptoms in preclinical models [223]. hiPSC-derived Schwann cells have been shown to be particularly sensitive to glucose-induced glucotoxicity, making them a valuable model for studying diabetic peripheral neuropathy (DPN) [220].

The hyperexcitability observed in ALS patients through clinical neurophysiological studies is successfully recapitulated in motor neurons derived from iPSCs of ALS patients with superoxide dismutase 1 (SOD1) and chromosome 9 open reading frame 72 protein (C9orf72) mutations; this hyperexcitability was lost in motor neurons generated from genetically corrected iPSC lines [224].

iPSC-derived cerebral organoids from Alzheimer’s disease (AD) patients show hallmark tau pathology, which is reduced by alpha and beta secretase inhibitors, thus providing a platform for testing anti-AD drugs [225]. Another study, utilizing iPSC-derived neuronal lines to investigate AD pathogenesis, concluded that enhancing mitophagy effectively reduces tau hyperphosphorylation associated with AD and improves memory deficits in tau-based models [226]. For AD, a 3D human neural cell culture system has been developed to replicate key events in its pathology [227].

IR significantly increases the risk of developing Parkinson’s disease (PD) by impairing neuronal metabolism, functionality, and survival. iPSC-derived midbrain organoids exposed to high insulin concentrations exhibited reduced dopaminergic neurons, increased oxidative stress, and decreased neural activity, suggesting IR as a key factor in PD pathogenesis and a potential target for preventing neurodegeneration [228].

The ability to generate large quantities of patient-specific neuronal cells from iPSCs provides a valuable platform for high-throughput screening of drugs and compounds that could ameliorate the effects of IR and diabetes on the nervous system. Functional readouts, such as neuronal excitability, synaptic transmission, and metabolic activity, can be used to evaluate the efficacy of candidate therapeutics as well as identify novel therapeutic targets and develop personalized treatment strategies for diabetic neuropathy.

A neural induction medium developed with physiological insulin and glucose levels allows modeling of gestational and type 1 diabetes (T1D), aiding in the study of early brain development and neurocognitive effects [229]. Recent studies reported regeneration of a type of glial cell, termed Müller glial cells (MGCs), as a treatment for retinal nerve damage and development. iPSC-derived MGCs were utilized therapeutically in glaucoma and retinitis pigmentosa, improving vision [230]. Transplanted iPSC-derived neural crest cells differentiated into Schwann cell-like and vascular cells [231]; these differentiated cell types induced paracrine action of growth factors, which may have therapeutic potential for conditions such as diabetic neuropathy. Overall, neuronal iPSCs can aid researchers in understanding disease mechanisms and be used for drug discovery and toxicity screening [232].

## 8. Limitations

iPSC models, while powerful, face several limitations. The field is still maturing, so iPSC-specific tools and expertise remain limited compared to other model systems. The low efficiency observed in iPSC reprogramming is largely due to the requirement for precise regulation and balanced expression of reprogramming factors, as well as the presence of multiple biological barriers [10,233,234]. Although most somatic cells are capable of initiating reprogramming, only a small subset ultimately achieves pluripotency. The previously held elite model—which posited that only rare, inherently predisposed cells could be reprogrammed—has been refuted [235]. Current evidence supports stochastic and deterministic models, suggesting that while all cells possess the potential for reprogramming, successful completion depends on random or tightly regulated mechanisms [236,237]. Furthermore, variables such as the type and differentiation status of donor cells, along with culture conditions, play a significant role in modulating reprogramming efficiency.

Pluripotent stem cells, including iPSCs and embryonic stem cells, hold significant therapeutic promise but are limited by their tumorigenic potential, notably the risk of teratoma formation due to their ability to differentiate into any cell type [238]. The propensity for tumor development varies depending on factors such as cell line, derivation method, number of transplanted cells, and site of injection, with genomic instability and viral integration contributing to oncogenic risks [239]. Strategies to mitigate these risks include using non-integrating or transgene-free reprogramming methods, omitting oncogenic factors like c-Myc, and applying small molecules or antibodies to selectively eliminate undifferentiated, tumorigenic cells prior to transplantation [240,241,242]. Despite these advances, challenges remain: Retroviral and lentiviral systems can cause insertional mutagenesis, and residual undifferentiated cells may persist even after differentiation, maintaining a risk for tumor formation [243]. Rigorous quality control—including sterility, genomic integrity, pluripotency marker assessment, and functional validation—is essential at every stage of clinical-grade iPSC production to ensure safety, consistency, and suitability for therapeutic use [244].

Although many iPSC lines representing different genetic disorders have been created, access and thorough characterization are often lacking, making them less useful for widespread research. There is a need for more mutant and reporter lines, ideally with isogenic controls, and for repositories to manage their distribution. Technical challenges include variability between iPSC lines in their phenotypic outcomes and the risk of accumulating genetic abnormalities during culture, which can be minimized with careful monitoring and use of early-passage cells. Variability can also arise from unnoticed genetic or epigenetic differences and inconsistent culture or differentiation protocols [245]. While initial concerns about the genetic instability of iPSCs have been largely addressed, iPSC-derived cells may not fully match their natural counterparts, often retaining some developmental immaturity. This immaturity, along with the component-focused nature of in vitro models, limits their ability to fully replicate disease processes seen in whole organisms [246]. Additionally, studying gene mutations is complicated by developmental effects, sometimes requiring advanced genetic engineering approaches to overcome these challenges.

Several guidelines, including those from the FDA (Food and Drug Administration), EMA (European Medicines Agency), and organizations like ISSCR (International Society for Stem Cell Research), regulate the clinical use of cells, tissue, and stem cell products [247]. Though broadly aligned, these guidelines may differ in specific details. Informed consent is required from both donors and recipients, with strict measures to protect genetic privacy and confidentiality. Donors should be clearly informed about how long their control over donated cells will last. All steps in iPSC-based therapies—from cell collection to transplantation—must follow Good Manufacturing Practice (GMP) standards, especially when genetic manipulation is involved, necessitating rigorous quality control. There are also significant ethical concerns regarding potential misuse of iPSCs, such as cloning, human–animal chimeras, or unauthorized gamete creation, which require ongoing oversight and regulation [248].

## 9. Conclusions

Human-induced pluripotent stem cells (iPSCs) have revolutionized the landscape of diabetes research and therapy by providing an unprecedented platform for disease modeling, drug discovery, and regenerative medicine [249]. Through advances in reprogramming and differentiation protocols, iPSCs can now be efficiently directed to generate functional insulin target cells, including hepatocytes, skeletal muscle cells, adipocytes, cardiomyocytes, and neuronal cells [8]. These iPSC-derived cells not only recapitulate key features of their in vivo counterparts but also enable the study of insulin resistance (IR) and diabetes pathogenesis in a patient-specific manner, overcoming limitations of traditional models. The ability to model IR in diverse tissues using iPSC-derived cells has deepened our understanding of the molecular mechanisms underlying type 2 diabetes and its complications. Given that insulin resistance underlies the development of type 2 diabetes, the roles and dysfunctions of key cellular components—hepatocytes, myocytes, and adipocytes—in its pathogenesis can be effectively investigated and modeled using induced pluripotent stem cell (iPSC) technologies. Furthermore, the development of three-dimensional cultures and organoid systems has enhanced the physiological relevance of in vitro models, paving the way for more accurate drug screening and toxicity testing. Importantly, the therapeutic potential of iPSC-derived insulin target cells is being realized, with advances in cell transplantation and tissue engineering offering promising avenues for restoring metabolic function in diabetic patients. Despite these achievements, several challenges remain. The functional maturation of iPSC-derived cells, the scalability of differentiation protocols, and ensuring safety and long-term integration post-transplantation require further optimization. Additionally, the cost and complexity of current protocols, as well as the need for standardized evaluation criteria, must be addressed to facilitate clinical translation.

In summary, iPSC technology holds immense promise for transforming the management of insulin resistance and diabetes. Continued interdisciplinary research and technological innovation will be essential to fully harness the potential of iPSC-derived insulin target cells for disease modeling, personalized medicine, and regenerative therapies, ultimately improving outcomes for individuals affected by diabetes worldwide.

## Figures and Tables

**Table 1:** Step-wise protocols for hepatocyte differentiation of iPSCs from their pluripotent origin to mature hepatocyte cells as outlined by several research groups.

Cell Origin	Culture Conditions	Protocol	Functional Analysis	Key Features of the Protocol	Reference
Human iPSC (Renal cells isolated from urine)	Small molecule-induced	Stage 1: Definitive endoderm: Days 1–3 Day 1: CHIR99021, Activin A Day 2–3: Activin A Stage 2: 4 days: Hepatic endoderm induction: DMEM-F12, 1%DMSO, KOSR, Doxycycline, 2-Mercaptoethanol, Glx Stage 3: HLC: days 12–15: DMEM F12, insulin, hepatocyte growth factor (HGF), dexamethasone (Dex), recombinant-human Oncostatin M (rhOSM209a.a), and forskolin	Indocyanine green (ICG) uptake Glycogen storage analysis Cytochrome P450 activity Urea production assay Real-time PCR Immunocytochemistry	Cost- and time-efficient 2D differentiation protocol, generating iPSC-derived HLCs within 18–20 days, Enhanced functionality and improved uniformity of cell morphology.	[39]
Human iPSC (Windy, K,FF-2(Commercial cell lines)	Small molecule induced	Stage 1: Endoderm induction: 5 days Activin A Stage 2: Subculturing of endoderm in 1% DMSO for 7 days, followed by Cos medium with HGF, Oncostatin-M, dexamethasone, and valproic acid for 7 days. The last 3 days: celecoxib. Stage 3: 3 days: Cos medium 004, HGF, Oncostatin M, dexamethasone, celecoxib. Stage 4: 4 days: Cos medium 004, celecoxib.	Real-time PCR Immunocytochemistry Bidirectional transporter assay	Hepatocytes derived from the endoderm exhibited increased gene expression of hepatocyte-specific markers and drug-metabolizing enzymes	[17]
BIONi010-C-CYP3A4–2TA-Nluc A reporter line containing a T2A-Nanoluciferase gene immediately upstream of the stop codon of the CYP3A4 gene, BIONi010 derived from Fibroblast of skin	Small molecule induced	Stage 1: Definitive endoderm: Days 1–3 Day 1: CHIR99021, Activin A Days 2–3: Activin A Stage 2: Hepatic Progenitor: 7-Day Reseeding: Progenitor Media, KOSR and DMSO Stage 3: Hepatocyte Maturation: 7 Days: Maturation Media, Dexamethasone, HGF, Oncostatin M, Hydrocortisone, Cholesterol Lipid Concentrate	Real-time PCR Immunocytochemistry In vitro toxicological studies Uptake and release of indocyanine green Periodic acid-Schiff staining for glycogen	An optimized protocol for differentiating hepatocyte-like cells (HLCs) from human iPSCs using both monolayer (2D) and suspension (3D) culture systems to enable organoid production.	[40]
iPSCs from urinary derived epithelial cells	Small molecule induced	Stage1: Definitive endoderm: Days 1–3 Day1: CHIR99021, Activin A Stage 2: Hepatic endoderm: 5 days: FGF, BMP-4, DMSO Stage 3: 5 days: Immature hepatocyte culture media: HGF, DMSO Stage 4: Mature hepatocyte culture media 10–12 days, HGF, DMSO, Oncostatin-M, dexamethasone	Real-time PCR Immunocytochemistry Western blotting	Protocol for differentiation of urine-derived iPSCs into hepatocytes and the upregulation of Cx32 enhances both the efficiency and maturity of this differentiation process	[41]
hCiPSCs human adult skin fibroblast and human adipose derived mesenchymal stromal cells	Small molecule induced	Stage 1 Definitive endoderm: 4 days: Activin A, BMP4, bFGF, Y27632, and CHIR99021 for 1 day in RPMI1640 medium with B27 supplement. Activin A, BMP4, Y27632, bFGF, and B27 supplement for another 3 days Foregut endoderm: 2 days: KGF, Y27632, and SB431542. hepatoblasts 1: 3 days: KGF, BMP4, BMP2, Y27632, and bFGF. Replating in hepatoblast 1 media with 10 μM Y27632 (Selleck, S1049) for 1 day. Hepatoblasts2: 3 days: William’s medium E, forskolin, EGF, and Y27632. hiHPC expansion media: DMEM/F12 mixed with William’s medium E in the ratio of 1:1 containing B27 supplement and forskolin, SB431542, EGF, CHIR99021, LP, dexamethasone and S1P, Nicotinamide, PVC, Heparin. hiHPCs maturation media: Williams’s medium E containing B27 supplement, forskolin, and SB431542.	PAS staining, LDA uptake and red O staining Real-time PCR Immunocytochemistry RNA sequencing	Differentiation protocol was established by replicating the two-stage development of hepatoblasts, enabling the efficient generation of hepatic progenitor cells from chemically induced pluripotent stem cells (hCiPSCs).	[42]

**Table 2:** Step-wise protocols for myogenic differentiation of iPSCs from their pluripotent origin to mature myogenic cells and fused myotubes as outlined by several research groups.

iPSC Origin	Type of Differentiation	Protocol	Culture Type	Key Features of the Protocol	Reference
HiPSC-AFR1	Small molecule induced	Stage 1: EB to myoblast (18 days): CHIR99021, the transforming growth factor-β (TGF-β) inhibitor SB431542, fibroblast growth factor-2 (FGF2), insulin growth factor-1 (IGF1), and heregulin-β-1 Stage 2: Myogenic amplification media (4–7 days): IGF1, FGF2, Heregulin-B-1 and Forskolin Stage 3: Myogenic maturation media (7 days)	EB+monolayer	High efficiency and purity in a short period Able to generate myotubes associated with a large pool of cell-cycle-arrested PAX7+ cells	[86]
*MYOD1*-hiPSCs	Small molecule induced	Stage 1: Myotube generation; Day 1: hESC medium without FGF-2 Day 3: Skeletal muscle induction media: αMEM supplemented with 10% KSR, 2% Ultroser G, and 2-ME. Stage 2: myotube maturation: Day 6–12 DMEM (high glucose, 1500 mg/L supplemented with 5% horse serum, recombinant human insulin-like growth factor 1 (IGF-1), and SB431542.	Monolayer	Expression of myogenic markers (MYOD1, MYOG, MYH3). Formation of multinucleated myotubes.	[87]
Human hiPSC (MiPS and BiPS)	Small molecule induced	Stage1: Day0–3 DICL (DMEM, ITS, CHIR, LDN) Stage 2: day 3–6 DICL+FGF Stage 3: Days 6–8 DK-LHIF medium (DMEM, KSR, LDN, HGF, IGF, FGF) Stage 4: Days 8–12 DKI (DMEM KSR, IGF) Stage 5: day12–30 DKI+ HGF Stage 6: (iMPCs): days 30–45 Skeletal muscle media(iMCs) Stage 7: days 45–60 terminal differentiation	Monolayer	Reprogramming into hiPSCs from primary muscle stem cells was found to be faster and 35 times more efficient than from blood cells	[88]
hPSC	Small molecule induced	Stage 1: mesoderm induction day1–4: MDM1-CHIR, SB, EGF, Insulin, dexamethasone Stage 2: Somite or myotome induction: Days 5–14 MDM2 LDN, SB, EGF, FGF, HGF, IGF-1 Stage 3: Cell sorting and expansion of myogenic progenitor: MDM 2 media Stage 4: Terminal differentiation: MDM3,15%KSR, IMDM, IGF	Monolayer	Efficient (45–65%) and short-term myogenic induction (two weeks) Using surface markers CD10+CD24, were able to purify skeletal myogenic progenitors from unwanted cells	[89]
Human iPSC	Small molecule induced	Primary differentiation: 3–4 weeks Proliferation: 1–2 days (Skeletal muscle growth media (SKGM)) Secondary differentiation: 1–2 weeks. KC (KSR/CHIR), KCTi (KSR/CHIR, TGF-β inhibitor SB431542), KCTiP (KSR/CHIR, TGF-β inhibitor SB431542, prednisolone)	Monolayer	Established and optimized a protocol to differentiate human-induced pluripotent stem cells (iPSCs) into late-stage myogenic cells	[90]
NSV44.1 and McA2.7	Small molecule induced	Stage 1: Primary Differentiation Day 0–Day 3: DiCL (DMEM-ITS-CHIR-LDN) Day 3–Day 6: DiCLF (DMEM-ITS-CHIR, LDN-FGF) Day 6–Day 8: DK-HiFL (DMEM, KSR, HGF, IGF, FGF, LDN) Day 8–Day 12: DK I (DMEM, KSR, IGF) Day 12–Day 30: DK-Hi (DMEM, KSR, HGF, IGF) Stage 2: Final Differentiation; 7–14 Days KCTiP (KSR/CHIR, TGF-β inhibitor SB431542, prednisolone)	Monolayer	Generated the first human iPSC-derived skeletal muscle model carrying the second most common PYGM mutation found in the Spanish population	[91]

**Table 3:** Stepwise protocols for adipocyte differentiation of iPSCs from their pluripotent origin to mature adipocyte cells as outlined by several research groups.

iPSC Origin	Method of Differentiation	Differentiation Media and Cocktail	Type of Adipocyte	Functional Analysis	Key Features of the Protocol	Reference
3T3-L1 embryonic fibroblastic cell line and a C3H10T1/2 mesenchymal stem cell line	Monolayer	3T3-L1 cell line Stage 1: 3days; DMEM, insulin, dexamethasone, isobutylmethylxanthine (IB), and Rosiglitazone. Day 4 DMEM and insulin Stage 2: Day 5–10; DMEM C3H10T1/2 Stage 1: DMEM, insulin, triiodothyronine, IBMX, dexamethasone, and indomethacin Stage 2: 4 days: DMEM, FBS, insulin, and T3	Brown	Oil Red O staining Gene expression analysis Male C57BL/6 mice were used as a model to investigate the function of miR-669a-5p in adipogenesis in vivo.	Expression of *miR-669a-5p* increases during the adipogenic differentiation of 3T3-L1 and C3H10T1/2 adipocytes.	[131]
HDFa-YK27-hiPSC human dermal fibroblast line and YK27-iPSC–derived iMSCs	Derivation of iMSCs from hiPSCs through embryoid bodies (EBs) formation	Stage 1: mesoderm induction: EB formation Stage 2: iMSC expansion- Stemline II, VEGF, BMP Stage 3: Preadipocyte growth: Preadipocyte basal media Stage 4: Adipocyte induction: DMEM, insulin, IBMX, dexamethasone and indomethacin	NA	Oil Red staining	Simple protocol eliminating the need for specialized equipment, expensive materials, or complex reagents Robust and cost-effective approaches to derive adipocytes and osteoblasts from iMSCs.	[132]
The human embryonic stem cell (hESCs) WA09, induced pluripotent stem cell (hiPSCs) line K3 and N4, generated from human neonatal foreskin fibroblasts	Direct differentiation in rotation culture from a Paraxial mesoderm (PM) precursor	Stage 1: Paraxial mesoderm induction: Day 1: hPSC MM media Day 2: hPSC CMM media Day3–4: Mesoderm induction media: DM, BMP4, bFGF, human IGF-I and rapamycin Day5–7 Paraxial mesoderm induction: DM (defined base medium), bFGF, human IGF-I, Rapamycin, WNT3, Noggin, (2′*Z*,3′*E*)-6-Bromoindirubin-3′-oxime (BIO) and forskolin. Stage 2: Brown adipocyte priming: BA1 media; DM bFGF, BMP7, human IGF-I, Y-27632 dihydrochloride, Rosiglitazone, Dexamethasone, T3 thyroid hormone, IBMX (3-Isobutyl-1-methylxanthine) and SB 431542 Stage 3: Brown adipocyte maturation BA2: BA2 is same as BA1 excluding SB 431542 and supplemented with Chemically Defined Lipid Concentrate.	Brown	Extracellular acidification rates (ECAR) Oxygen consumption rate (OCR) assay, Lipolysis assay, Transplantation of BAs into immunocompromised (non-obese diabetic/severe combined immunodeficiency [NOD/SCID]) mice fed on a regular chow diet	Highly efficient generation of BAs through a paraxial mesoderm progenitor state	[133]
hESC lines (H1 and H9)	MSC through EB in a retinoic acid-based method	Stage 1: EB formation; Day 0–7. DMEM+ RA Stage 2: differentiation into MSC; Days 7–12; Differentiation media Stage 3: MSC expansion; Differentiation media and bFGF, Stage 4: Adipogenic differentiation Protocol 1 (Pr1): knockout DMEM-F12, KSR, 3-isobutyl-1-methylxanthine (IBMX), dexamethasone, insulin, indomethacin and pioglitazone. Protocol 2 (Pr2), MEM-alpha, IBMX, dexamethasone, insulin, indomethacin and Roziglitazone	NA	Oil Red O and BODIPY staining, Adipogenesis marker expression (FABP4, PPAR γ, and adiponectin) by immunocytochemistry Flow cytometry, RNA sequencing	Production of abundant multipotent MSCs, which can be expanded through multiple passages in culture. These MSCs possess a strong potential for further differentiation into adipocytes	[134]
hiPSC lines reprogrammed from fibroblasts	Monolayer	Stage 1: Mesoderm differentiation; Day 0–4; STEM Pro34, Glutamax, Ascorbic acid, BMP-4, Activin A. Stage 2: Adipocyte differentiation; Days 5–10; DMEM/F12, insulin, methylxanthine (IBMX), dexamethasone, and indomethacin Stage3: Adipocyte maturation; Day 10–20: DMEM/F12, Insulin	Beige	O2 consumption measurement Transcriptome analysis Transplantation of adipocytes by subcutaneous injection in the back of 6-week-old FoxN1^Nu^ athymic mice	A highly efficient and scalable protocol for differentiating hiPSCs into beige adipocytes through sequential mesodermal and adipogenic induction steps.	[135]
Human iPSC	Monolayer, through MSC to adipocyte precursor and to adipocytes	Stage 1: Mesoderm Induction Days 0–5: MIM. Stage 2: Generation of MSC from mesoderm; Days 5–12 MesenCult-ACF Plus medium Stage 3: Beige adipogenic precursor induction; Days 0–2, MesenCult-ACF, SB 431542, IL-4. Stage 4: Beige adipocyte induction media: days 2–5; insulin, T3 Roziglitazone, isobutylmethylxanthine (IBMX), dexamethasone, indomethacin, SB 431542, EGM-2 Stage 5: Beige adipocyte maintenance; Days 5–14: EGM-2, SB 431542, insulin, T3, Roziglitazone,	Beige	O2 consumption measurement Glucose uptake assay Fatty acid uptake assay Flow cytometry qRT-PCR Immunoblotting JC-1 assay Mass spectrometry	A stepwise approach for deriving highly expandable mural-like MSCs from iPSCs, converting them into adipogenic precursors, and subsequently differentiating them into beige adipocytes	[136]

**Table 4:** Stepwise protocols for cardiomyocyte differentiation of iPSCs from their pluripotent origin to mature cardiomyocyte cells, as outlined by several research groups.

Cell Origin	Type of Differentiation	Protocol	Functional Analysis	Key Features of the Protocol	References
HiPSC (SCVI-273, SCVI-114, SCVI-202, and SCVI-111) All are commercial cell line derived from peripheral blood	Small molecule induced	Stage 1: 3 days: Day 1; CHIR99021 RPMI/B27-insulin, Day 2–3 RPMI/B27-insulin Stage 2: Day 3 Combined media and IWP2 Day5: RPMI/B27-insulin Day7: RPMI/B27-insulin every 3 days	Contractility measurements and calcium transient analysis Immunofluorescence qRT-PCR	Directed hPSCs to cardiomyocytes in a serum-free, defined system via temporal regulation of canonical Wnt signaling pathways	[164] [176]
WTC-11 WTC-Cas9 (generated by inserting CAG-rtTA::TetO-Cas9 in WTC-11) derived from skin fibroblasts	Small molecule induced	Stage 1: 2 days: CHIR99021 Stage 2: 2 days: IWR1 Days 7–15: Basic media with insulin	RNA sequencing, Ca^2+^ and voltage imaging, Morphological analysis, Mitochondrial function analysis	Reproducible, scalable, and resource-efficient approaches to generate iPSC-CMs	[177]
H1 (WA01), H9 (WA09) mND2–0 (embryonic stem cells)	Small molecule induced	Stage 1: mesoendoderm induction: Day 0: CDM-A: Cardiac Differentiation Basal Medium (CDBM) + CHIR99021 Day 1; CDM-B: CDBM + heparin Stage 2: Cardiac progenitor induction: Day 2–4: CDM-C-: CDBM + heparin + IWP2 Stage3: Cardiac differentiation: Day 5–6: CDM-B Stage 4: Cardiomyocyte maturation Day 7: CDM-D-: CDBM + Insulin	Immunofluorescence Flow cytometry	A cost-effective method to derive cardiomyocytes from human pluripotent stem cells High-purity beating cardiomyocytes can be observed within 7 days of differentiation	[178]
Human Ips Cells (derived from skin fibroblast)	Small molecule induced	Stage 1: 1 day: CHIR99021 Stage 2: days 2: WntC59, XAV939, human Sfrp2, human Wnt3a protein Stage 3: Day 5–9: differentiation media (RPMI1640+ Ascorbic acid) Day 9–14: RPMI1640+ B27	Immunofluorescence staining Sarcomere analysis Patch clamp analysis	They have replaced broad-spectrum pharmacological inhibitors with Sfrp2, which gave rise to mature cardiomyocytes, as evidenced by their sarcomere structure, electrophysiological profiles, and ability to form gap junctions	[172]
H1, H9 (Embryonic stem cells), iPSC reprogrammed from fibroblast)	Small molecule induced	Stage 1: 1 day: CHIR9902 Days 2–3: RPMI1640 with B27 without insulin Stage 2: 2 days: IWP2 Stage 3: 2 days: RPMI1640 with B27 without insulin Day 7: RPMI-B27 with insulin	Immunocytochemistry, Flowcytometry, Microelectrode array measurements, Electrophysiological parameter analysis	Developed a 96-well microplate-based protocol for differentiating human pluripotent stem cells into functional cardiomyocytes	[179]

**Table 5:** Stepwise protocols for neuronal differentiation of iPSCs from their pluripotent origin to mature neuronal cells as outlined by several research groups.

Cell Origin	Culture Conditions	Protocol	Functional Analysis	Key Features of the Protocol	Reference
WT1and WT2 (fibroblast derived cell line) and WT2 (Commercial cell line)	STEMdiff^™^ Neural System	Stage 1: Neural induction: 9 days: Neural induction media with SMADi and 10 µM Y-27632 Stage 2: subculturing Stage 3: generation of neuronal precursor: 7 days: Neural differentiation media Stage 4: Maturation: Neural maturation media	Immunocytochemistry RNA sequencing	Identified key genes within the human neuronal differentiation network—novel candidates that are likely to play crucial roles in neurogenesis	[210]
HiPSC Coriell ND41865 (commercial cell line derived from skin fibroblast)	Small molecule induced	Differentiation media 1: SB431542, LDN193189, DMH-1, and recombinant human DKK-1 protein Differentiation media 2: SB431542, LDN193189, DMH-1, and cyclopamine Differentiation media 3: Neurobasal media with 1X Glutamax,1X N-2 supplement, 1X B27 without Vitamin A, BDNF, GDNF, Ascorbic Acid, cAMP, Laminin, and 1X Antimycotic-Antibiotic.	Immunocytochemistry Whole-cell patch clamping, Flow cytometry	This study developed a phenotypic model of hiPSC-derived cortical neurons, characterized their maturation process, and investigated its application for disease modeling with the integration of multi-electrode array (MEA) technology.	[207]
H9 hESC (embryonic stem cell line), L2122mutation in *PINK1 (PTEN-induced kinase)* gene, and L2131 (familial control of *PINK1* mutated gene hiPSC lines derived from skin fibroblast)	Small molecule induced	Stage 1: LDN193189 and SB431542 and 2μM each of purmorphamine and RA Stage 2: NB/B27 medium supplemented with BDNF.	Immunocytochemistry Real-time quantitative PCR Electrophysiology	A novel approach was introduced for deriving hindbrain 5-HT neurons from hPSCs through ventral neural progenitor formation and stimulation of hindbrain serotonergic differentiation	[211]
hiPSC (WTC-11 (Commercial cell line derived from skin fibroblast))	Small molecule induced	Stage1: Days 0–6: CHIR99021, DMH-1 and SB431542 Stage 2: days 6–12: Stage 1 media with RA and Pur Stage 3: RA and Pur Stage 4: RA, Pur, CpdE, IGF-1, BDNF, and CNTF	qRT-PCR Functional analysis on microelectrode array Immunocytochemistry	They characterized the functionality of iPSC-derived MNs via electrophysiological analysis of neuronal network connectivity.	[212]
hiPSC (H9 and MEL1 (Commercial Embryonic stem cell lines), and J1 human induced iPSC derived from fibroblast cells)	Small molecule induced	Stage 1: L-glutamine, SHH C25II, LDN, B431542, CHIR99021 (from day4 different concentration of CHIR), and Rock inhibitor (Y-27632) Stage 2: Neurobasal/B27/L-Glu supplemented with BDNF (brain-derived neurotrophic factor, ascorbic acid, GDNF (glial cell line-derived neurotrophic factor, TGFβ3 (transforming growth factor type β3, dibutyryl cAMP, and CHIR Stage3: NB/B27/L-Glu, BDNF, ascorbic acid, GDNF, dbcAMP, and TGFβ3 until day 16, with adding DAPT	Western blotting, RNA sequencing Electrophysiological measurements	Developed a two-step WNT signaling activation strategy that improves expression of midbrain markers, such as Engrailed-1 (EN1), while minimizing expression of contaminating posterior (hindbrain) and anterior (diencephalic) lineage markers	[213]

## Data Availability

No novel data was generated in the writing of this review article.
